# Supplementary material for: MELAS-Derived Neurons Functionally Improve by Mitochondrial Transfer from Highly Purified Mesenchymal Stem Cells (REC)
Source: Int J Mol Sci. 2023 Dec 6;24(24):17186. doi: 10.3390/ijms242417186 (PMC10742994; doi:10.3390/ijms242417186)
Supplement: Supplementary file 1 [file ijms-24-17186-s001.zip › ijms-2751408-supplementary.pdf]

Supplementary Figure S1

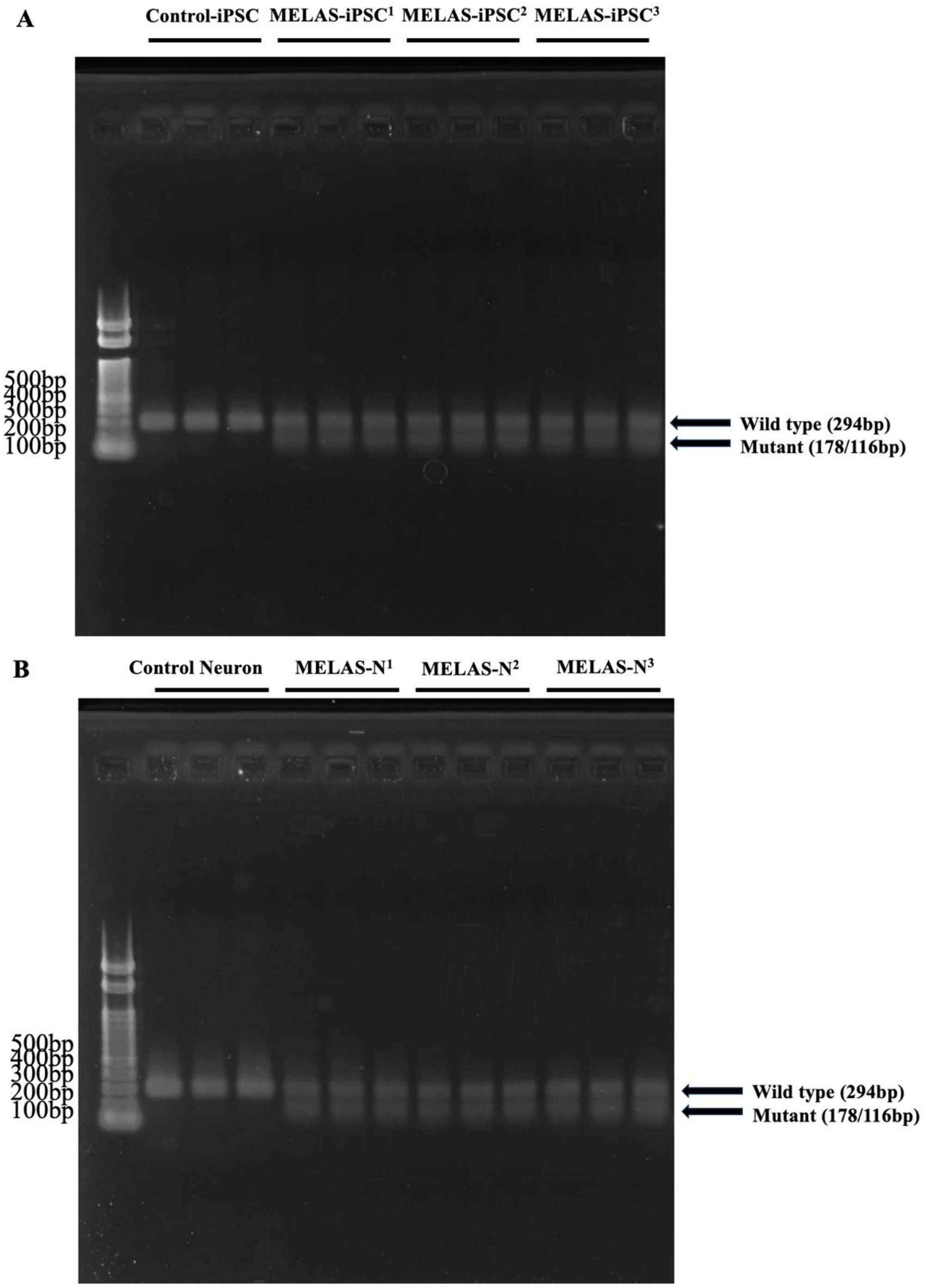

Figure S1. RFLP analysis of the m.3243A>G heteroplasmic mutation.

(**A**) Electropherogram showing the heteroplasmy level of m.3243A>G mutation in each group of iPSC cells. (**B**)Electropherogram showing the heteroplasmylevel of m.3243A>G mutation in neurons of each group. The wild-type mtDNA amplified a fragment of 294 bp. In the presence of the m.3243A>G mutation, the PCR product was cleaved by ApaI restriction endonuclease into two fragments of 178 and 116 bp.
